# Supplementary material for: Genomic attributes of Vibrio cholerae O1 responsible for 2022 massive cholera outbreak in Bangladesh
Source: Nat Commun. 2023 Mar 1;14:1154. doi: 10.1038/s41467-023-36687-7 (PMC9977884; doi:10.1038/s41467-023-36687-7)
Supplement: Supplementary file 1 — Supplementary Information [file 41467_2023_36687_MOESM1_ESM.pdf]

# Supplementary Information for

# Genomic attributes of *Vibrio cholerae* O1 responsible for 2022 massive cholera outbreak in Bangladesh

**Authors**:

Md Mamun Monir[1], Mohammad Tarequl Islam[1], Razib Mazumder[1], Dinesh Mondal[1], Kazi Sumaita Nahar[1], Marzia Sultana[1], Masatomo Morita[2], Makoto Ohnishi[2], Anwar Huq[3], Haruo Watanabe[2], Firdausi Qadri[1], Mustafizur Rahman[1], Nicholas Thomson[4], Kimberley Seed[6], Rita R. Colwell[3,5], Tahmeed Ahmed[1], and Munirul Alam[1§]

**Authors affiliations**:

*1. icddr, b, (International Centre for Diarrhoeal Disease Research, Bangladesh), Dhaka, Bangladesh*

*2. National Institutes of Infectious Diseases (NIID), Tokyo, Japan*

*3. Maryland Pathogen Research Institute, University of Maryland, USA*

*4. Sanger Institute, Cambridge, UK*

*5. Johns Hopkins Bloomberg School of Public Health, Baltimore, Maryland, USA*

*6. University of California, Berkeley, USA*

[§]**Corresponding author**: Munirul Alam, PhD
Mailing address:
Infectious Diseases Division (IDD)
International Centre for Diarrheal Disease Research, Bangladesh (icddr,b)
68, Shaheed Tajuddin Ahmed Sarani,
Mohakhali, Dhaka 1212,
Bangladesh
Tel: +88-02-9840523-32 Ext. 2433/2490
Fax: +88-02-8812529
E-Mail: munirul@icddrb.org

## Supplementary methods

### SNP calling

Burrows-Wheeler Alignment tool (BWA)[1] was used to align high-quality reads with reference genome sequence of *V. cholerae* N16961 El Tor (NCBI Accession ID: NC_002505.1 and NC_002506.1) for variant calling. Tool for sequence alignment/map (Samtools v1.9)[2] and Bcftools v1.9[3] were used to call genome variants.

### Typing integrating conjugative element

Nucleotide blast command line applications (blastn v2.13.0)[4] was used to match contigs of the strains with seven publicly available sequences of the Integrative and conjugative elements (ICEs)- ICEVchban5 (GQ463140.1), ICEVchind4 (GQ463141.1), ICEVchind5 (GQ463142.1), ICEVchmex1 (GQ463143.1), ICEVflInd1 (GQ463144.1), $ICE^{TET}$ (MK165649.1), and $ICE^{GEN}$ (MK165650.1). Then in-house R-script was used for summarizing the blast results and tabulating best matched ICEs for the strains.

## Supplementary figures

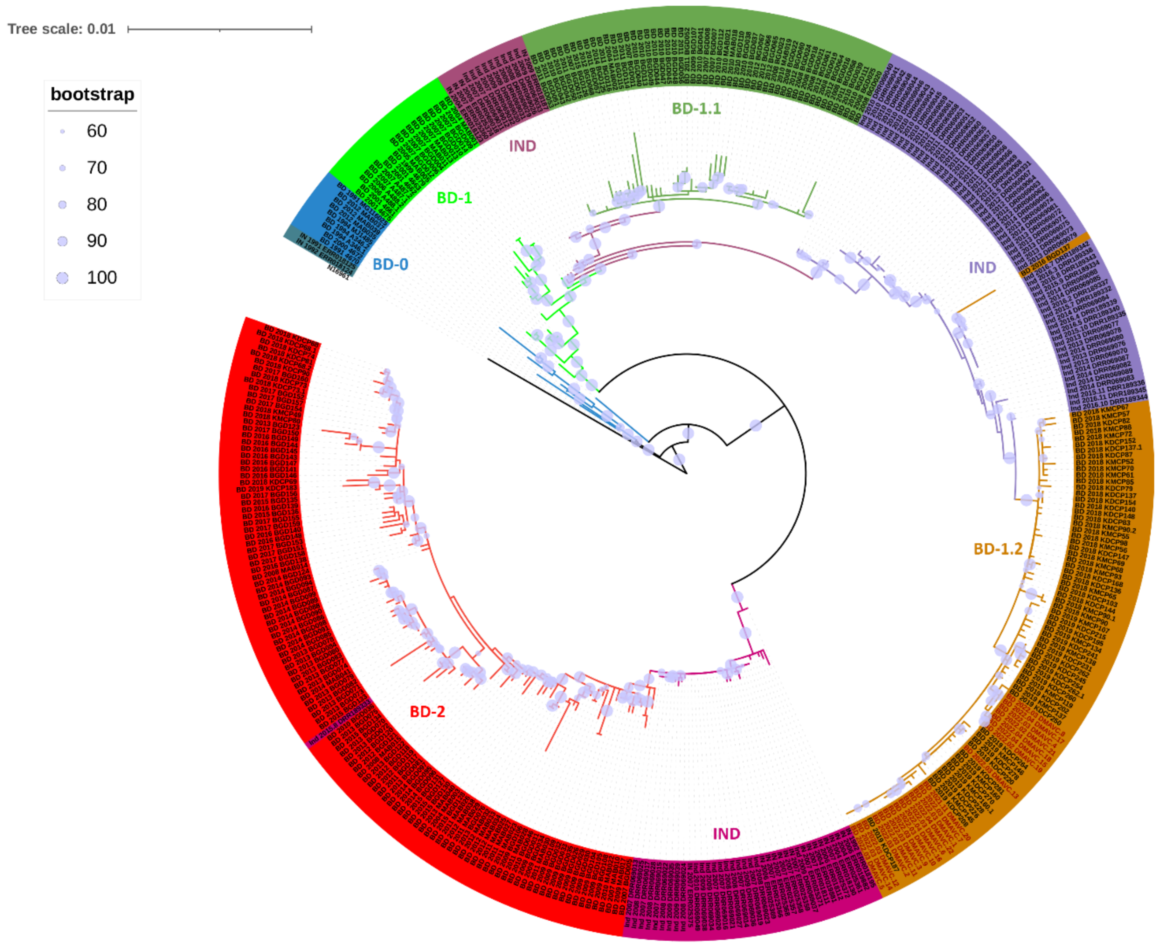


**Supplementary Figure 1: Phylogenetic clusters of the *V. cholerae* strains isolated from Bangladesh and India.** The greater than 60% bootstrap support value is shown by using a filled circle on the tree. Recently emerged BD-1.2 strains are suggested to have as most recent common ancestor with the strains isolated from India, i.e., predominant in India when BD-2 strains were predominant in Bangladesh. The 22 outbreak strains sequenced in the present study were colored in red, and all belonged to BD-1.2 subclade. Genetic variant data used to construct phylogenetic tree are provided in Source Data 1 and related metadata in Supplementary Data 1.

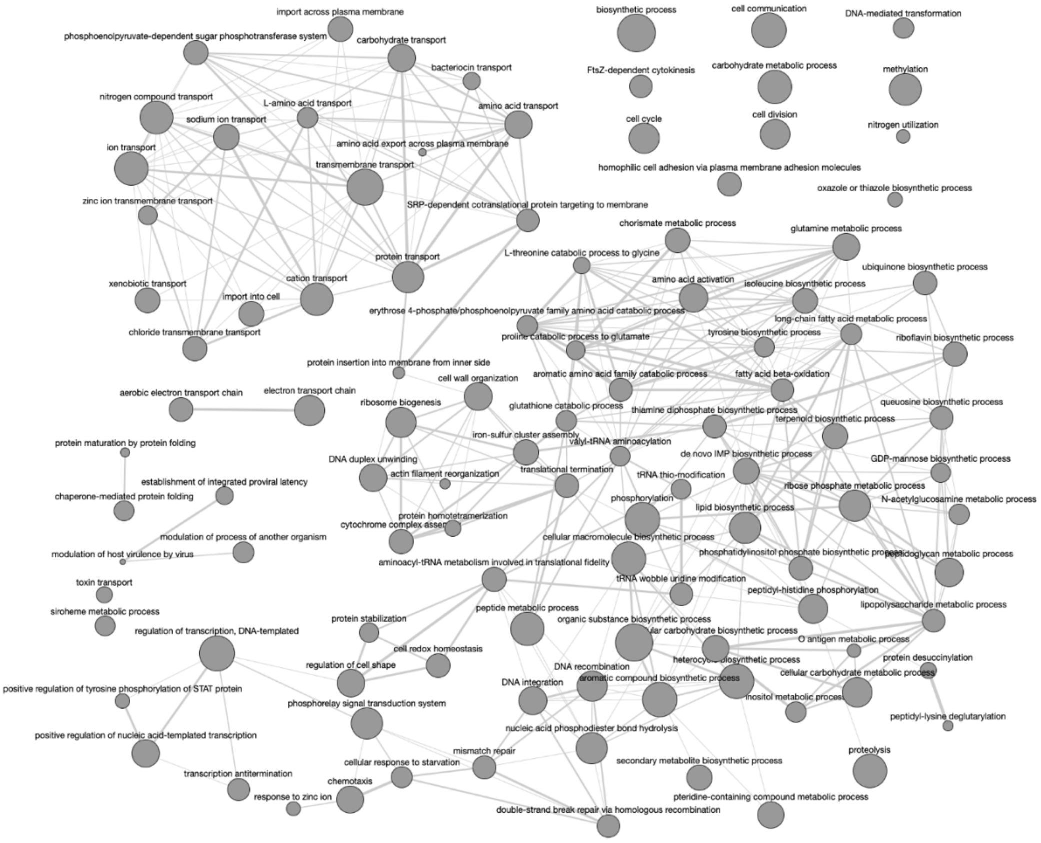


**Supplementary Figure 2: Biological process network of lineage associated mutant genes.** Complex network of the biological process was found using REVIGO. Ontology of genes were obtained using PANNZER. The GO terms frequencies in the underlying gene ontology annotation are indicated by the bubble size. Highly similar GO terms are connected by edges and the line width indicates the degree of similarity. Gene ontology terms and related descriptions are provided as a Source Data file 4.

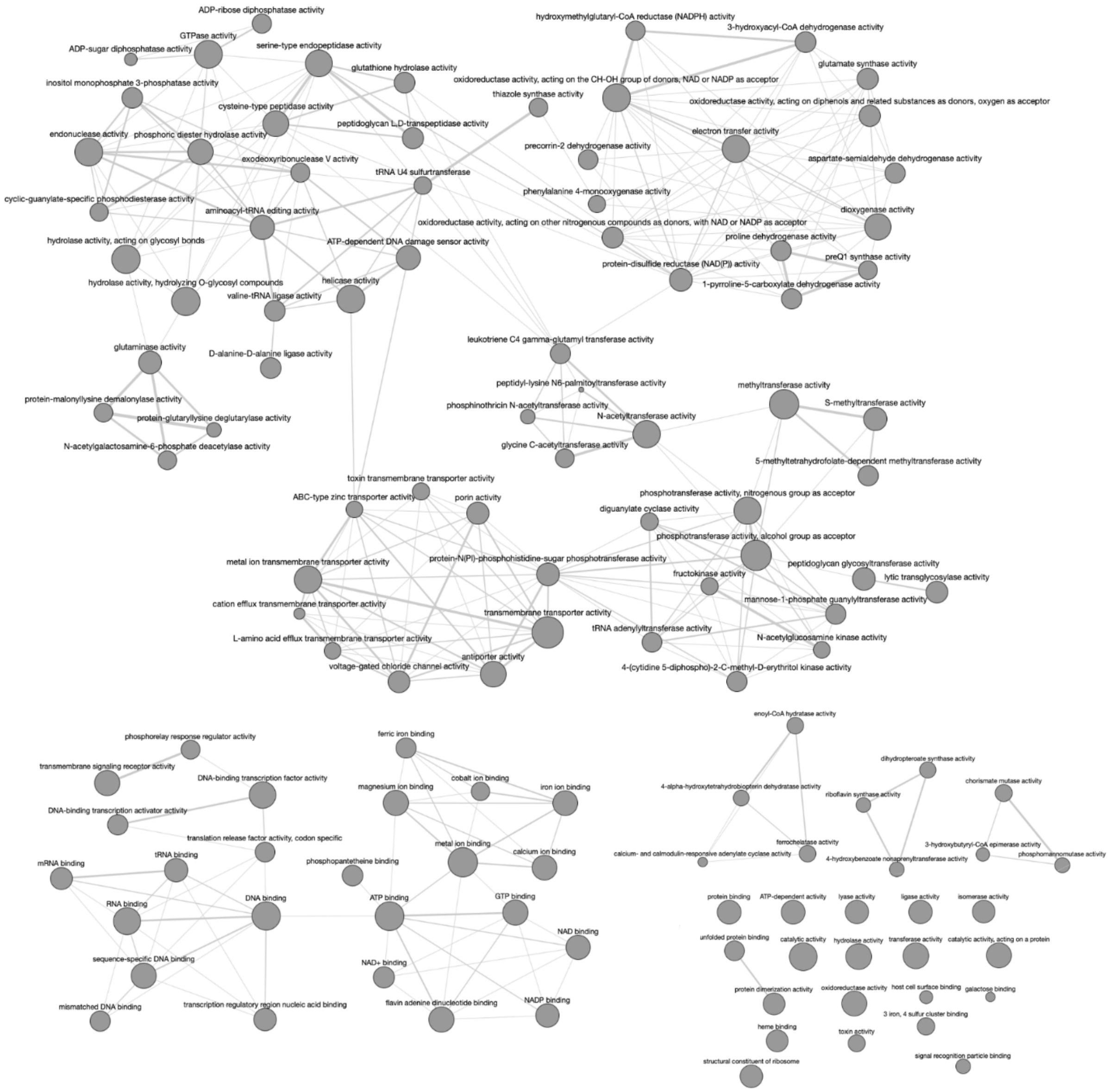


**Supplementary Figure 3: Molecular functions (MF) network of lineage associated mutant genes.** Complex network of important MF was obtained after summarizing the GO terms obtained from PANNZER, that suggest evolution of the bacterium though mutation in the key genes may be fundamental for their fitness and adaptation. Gene ontology terms and related descriptions are provided as a Source Data file 4.

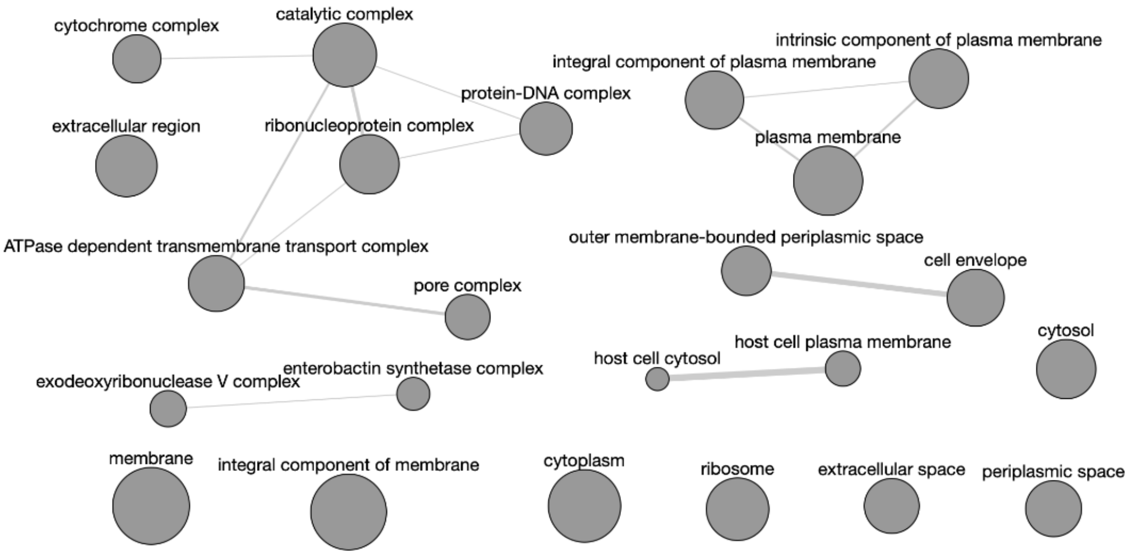


**Supplementary Figure 4: Cellular locations of lineage associated mutant gene products.** Products of mutant genes locates in cytoplasm, ribosome, plasma membrane, extracellular region, host cell plasma membrane, host cell cytosol, etc. Gene ontology terms and related descriptions are provided as a Source Data file 4.

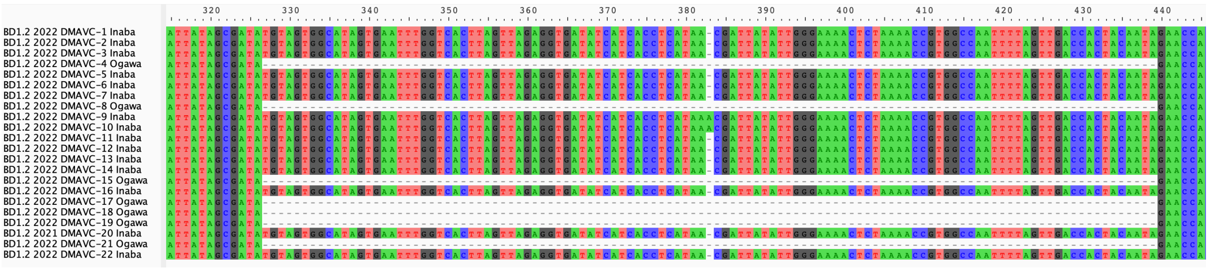


**Supplementary Figure 5: Partial view of *rfbT* gene alignment for the sequenced strains.** Within 21 strains isolated in 2022, 7 were Ogawa and 14 were Inaba. All of the Inaba strains had this insertion (2 strains had 113bp insertion and remains 12 had 112bp insertion). In addition, an Inaba strain isolated in 2021 also had 112bp insertion in *rfbT* gene.

# Supplementary Tables

**Supplementary Table 1.** Allelic missense SNP differences between BD-1.1 and BD-1.2 strains

| SNP | REF | ALT | Freq BD1.1 | Freq BD1.2 | Locus tag | Gene | AA change | Product |
|---|---|---|---|---|---|---|---|---|
| S1_64791 | C | T | 0 | 84 | VC_0065 | thiG | Ala191Val | Thiazole synthase |
| S1_93723 | G | A | 0 | 84 | VC_0094 | ubiA | Thr199Ile | 4-dydroxybenzoate octaprenyltransferase |
| S1_148860 | C | T | 0 | 84 | VC_0157 | | Cys45Tyr | S8 family peptidase |
| S1_674880 | C | T | 0 | 84 | VC_0633 | ompU | Gly325Asp | Porin OmpU |
| S1_1068347 | G | A | 0 | 84 | VC_0998 | | Ala1103Thr | Hypothetical protein |
| S1_1388812 | C | T | 0 | 84 | VC_1306 | | Ala447Val | YcjX family protein |
| S1_1404113 | C | T | 0 | 84 | VC_1320 | rstA | Asp89Asn | Transcriptional regulatory protein |
| S1_1551891 | C | G | 0 | 54 | VC_1451 | rtxA | Ala582Gly | MARTX multifunctional-autoprocessing repeats-in-toxin holotoxin RtxA |
| S1_1882714 | G | A | 0 | 84 | VC_1748 | | Ser48Asn | Hypothetical protein |
| S1_2098531 | C | A | 0 | 84 | VC_1949 | | His34Asn | L-tyrosine/L-tryptophan isonitrile synthase |
| S1_2391706 | G | A | 0 | 84 | VC_2237 | mltD | Gly246Asp | Membrane-bound lytic murein transglycosylase D precursor |
| S1_2405951 | A | C | 0 | 84 | VC_2251 | Skp | Leu46Trp | Chaperone protein Skp precursor |
| S1_2466598 | T | C | 0 | 84 | VC_2319 | recD | Tyr680Cys | exodeoxyribonuclease V subunit alpha |
| S1_2577786 | C | T | 0 | 84 | VC_2407 | | Arg491His | penicillin-binding protein 3 |
| S1_2873743 | C | T | 0 | 84 | VC_2701 | dsbD | Thr583Ile | protein-disulfide reductase DsbD |
| S2_398116 | G | T | 0 | 84 | VC_A0442 | aacA4 | Gly29Cys | GNAT family N-acetyltransferase |
| S2_460594 | C | T | 0 | 84 | VC_A0526 | clcA | Gly104Glu | H(+)/Cl(-) exchange transporter ClcA |
| S2_577065 | C | A | 0 | 84 | VC_A0640 | | Ala121Ser | YeeE/YedE family protein |
| S2_968585 | G | A | 0 | 84 | VC_A1020 | gltR_3 | Ala174Val | HTH-type transcriptional regulator GltR |
| S1_831779 | C | A | 53 | 0 | VC_0775 | vibH | Ala172Ser | norspermidine-2,3-dihydroxybenzoate synthase VibH |
| S1_1117838 | T | G | 53 | 0 | VC_1047 | fadJ | Val546Gly | fatty acid oxidation complex subunit alpha FadJ |
| S1_1354024 | G | A | 53 | 0 | VC_1279 | opuD | Ala136Thr | BCCT family transporter |
| S1_2085882 | C | T | 53 | 0 | VC_1934 | cph2_6 | Ala340Val | Phytochrome-like protein cph2 |
| S1_2191730 | A | G | 53 | 0 | VC_2036 | asd1 | Val347Ala | aspartate-semialdehyde dehydrogenase |
| S1_2240207 | C | T | 53 | 0 | VC_2080 | cdhR | Gly52Arg | AraC family transcriptional regulator |
| S1_2476906 | G | A | 53 | 0 | VC_2323 | tehA | Ala91Val | Tellurite resistance protein TehA |
| S1_2485904 | G | A | 53 | 0 | VC_2335 | | Ala158Thr | gamma-glutamylcyclotransferase |
| S2_568477 | C | T | 53 | 0 | VC_A0631 | | Arg106His | GNAT family N-acetyltransferase |
| S2_609892 | G | A | 53 | 0 | VC_A0675 | narQ | Arg532Cys | nitrate/nitrite two-component system sensor histidine kinase NarQ |

SNPs were labeled according to chromosome name and base pair position. REF = Reference allele, and ALT = alternative allele of the SNPs. Freq_BD-1.1 refers to the frequency BD-1.1 strains were isolated between 2004-2015, and Freq_BD-1.2 refers to the frequency of BD-1.2 strains isolated between 2018-2019. Locus tag are identifiers systematically applied to gene of reference genome N16961 (NCBI database). AA change refer amino acid change.

**Supplementary Table 2.** Allelic differences in synonymous and intergenic SNPs between BD-1.1 and BD-1.2 strains

| SNP/indel | REF | ALT | Freq BD-1.1 | Freq BD-1.2 | Type | Genes | Nt changes |
|---|---|---|---|---|---|---|---|
| S1_139591 | T | C | 0 | 84 | synonymous | ftsX | c.750T>C |
| S1_454734 | G | A | 0 | 84 | upstream | arcA_1 | c.-571C>T |
| S1_683129 | G | C | 0 | 84 | upstream | rimP | c.-222G>C |
| S1_953331 | T | G | 0 | 84 | synonymous | thiI | c.390T>G |
| S1_967866 | A | G | 0 | 84 | upstream | queF | c.-4229T>C |
| S1_1429844 | T | C | 0 | 84 | synonymous | rppH_2 | c.1425T>C |
| S1_1981925 | G | A | 0 | 84 | synonymous | cydB_1 | c.909C>T |
| S1_2002816 | G | A | 0 | 84 | synonymous | argT | c.660C>T |
| S1_2332052 | T | C | 0 | 84 | downstream | ispE | c.*4260T>C |
| S1_2484852 | C | T | 0 | 84 | synonymous | zntB | c.381C>T |
| S2_350647 | G | A | 0 | 84 | synonymous | PROKKA_03031 | c.252G>A |
| S2_820659 | C | T | 0 | 84 | synonymous | PROKKA_03482 | c.157C>T |
| S1_1812498 | C | T | 53 | 0 | upstream | czcA | c.-3593G>A |
| S1_1970973 | A | G | 53 | 0 | synonymous | alaE | c.183T>C |

SNPs were labeled according to chromosomal position. For example, "S1_1905668" is an SNP/indel site, where "S" stands for site and "1905668" stands for location of the site base pair. REF = reference allele; and ALT = alternative allele. Freq_BD1.1 = frequency of the BD-1.1 strains isolated between 2004-2015, and Freq_BD1.2 = frequency of the BD-1.2 strains isolated between 2016-2022. Type = type of variant. AA_change = amino acid change.

**Supplementary Table 3.** Allelic indel differences between BD-1.1 and BD-1.2 strains

| SNP/indel | REF | ALT | Freq BD-1.1 | Freq BD-1.2 | Type | Genes | Nt changes | AA changes |
|---|---|---|---|---|---|---|---|---|
| S1_888713 | ATTT TTTTT | ATTTTTT TTTT | 0 | 84 | upstream | ctpH_1 | 317-316 ins A | NA |
| S1_890392 | TAAA AAA | TAAAAAA AA | 0 | 84 | upstream | ctpH_1 | 1994-1993 ins T | NA |
| S1_2558670 | TCCC CC | TCCCCCC C | 0 | 84 | upstream | carB | 1397-1396 ins G | NA |
| S2_604530 | AGAA CGAA GATT GC | AC | 0 | 84 | Disruptive in-frame deletion | sglT | 572-583 del CAATCTTCGTTC | Ala191_Leu 195delinsVa l |
| S1_1154205 | GAAC TAT CTCC ATA ACAA ACT ATCT CCA TAA | GAACTA T CTCCAT AA | 53 | 0 | frameshift | cph2_3 | 1547-1562 del CAAACTATCTCCATAA | Leu516fs |
| S2_990917 | GCCC C | GCCC | 53 | 0 | frameshift | glnP | 650 del C | Pro217fs |
| S1_2961141 | CGAT CAAG G | CGATCA A GGAGG GT CATTAA AT ATATAT AA AGATCA AG G, CGATCA AGGAG GG TCATTA AA TATATA T AAAGAT CTATAT A GAGATC AAGG | 51 | 0 | Feature elongation | NA | NA | NA |
| S1_2961143 | ATCA AGG | ATCAAG G AGGGTC A TTAAAT A TATATA AAGATC T ATATAG A GATCTT T TCAAGG , ATCAAG G | 14 | 0 | Feature elongation | NA | NA | NA |

| | | | | | | | | |
|---|---|---|---|---|---|---|---|---|
| | | AGGGT CAAGG | | | | | | |

Insertion-deletions (indels) were named according to chromosomal positions. For example, "S1_1905668" is an SNP/indel site, where "S" stands for site and "1905668" stands for location of the site base pair. REF = reference allele; and ALT = alternative allele. Freq_BD1.1 = frequency of BD-1.1 strains isolated between 2004-2015, and Freq_BD1.2 = frequency of BD-1.2 strains isolated between 2016-2022. Type = type of variant. Nt changes = Nucleotide changes in the protein coding region of a gene, and AA change = amino acid changes.

**Supplementary Table 4.** Antibiotic resistance gene frequency for global and Asian clade lineages

| AMR gene | BD-1 n=20 | AS1 n=18 | T11 n=5 | IND-1 n=15 | BD-1.1 n=51 | IND-1.1 n=11 | T12 n=7 | LAT-3 n=7 | IND-1.2 n=44 | T13 n=24 | IND-1.3 n=8 | BD-1.2 n=79 | AS-2 n=59 | IND-2 n=9 | BD-2 n=122 |
|---|---|---|---|---|---|---|---|---|---|---|---|---|---|---|---|
| varG | 20 | 17 | 5 | 15 | 51 | 11 | 7 | 7 | 44 | 24 | 8 | 79 | 59 | 9 | 122 |
| catB9 | 20 | 18 | 5 | 15 | 51 | 11 | 7 | 7 | 44 | 24 | 8 | 79 | 59 | 9 | 118 |
| dfrA1 | 20 | 18 | 5 | 15 | 51 | 11 | 7 | 7 | 44 | 24 | 8 | 79 | 56 | 7 | 112 |
| aph(6)-Id | 18 | 18 | 5 | 15 | 51 | 11 | 7 | 7 | 31 | 0 | 8 | 79 | 54 | 7 | 108 |
| aph(3")-Ib | 18 | 16 | 5 | 15 | 51 | 11 | 7 | 7 | 31 | 0 | 8 | 79 | 53 | 7 | 108 |
| sul2 | 17 | 15 | 5 | 15 | 51 | 11 | 7 | 7 | 31 | 0 | 8 | 79 | 54 | 7 | 108 |
| floR | 18 | 14 | 5 | 15 | 51 | 11 | 7 | 7 | 31 | 0 | 8 | 79 | 12 | 0 | 0 |
| tetA(D) | 0 | 2 | 0 | 0 | 0 | 0 | 0 | 0 | 0 | 0 | 0 | 0 | 42 | 7 | 108 |
| dfrA31 | 0 | 0 | 0 | 0 | 0 | 0 | 0 | 0 | 0 | 0 | 0 | 0 | 27 | 4 | 21 |
| qnrVC1 | 0 | 0 | 0 | 0 | 0 | 0 | 0 | 0 | 0 | 0 | 0 | 0 | 27 | 4 | 21 |

Here, AMR gene = antimicrobial resistance gene; n = number of strains of the lineages. BD-1, BD-1.1, BD-1.2, and BD-2 are the lineages/groups of strains isolated from Bangladesh; IND-1, IND-1.2, IND-1.3, and IND-2 are the groups of strains isolated from India; AS-1 and AS-2 are groups of the strains isolated from multiple Asian countries; T11, T12, and T13 are African transmission lineages; and LAT-3 is the Latin American transmission lineage.

**Supplementary Table 5.** Serotypes of Bangladesh strains isolated between April 2017 and March 2022

| Year | Month | Total tested strains | Inaba | Ogawa |
|---|---|---|---|---|
| 2017 | April | 3 | 3 | |
| | May | 19 | 19 | |
| | June | 2 | 2 | |
| | August | 1 | 1 | |
| | September | 3 | 3 | |
| | October | 2 | 2 | |
| | November | 1 | 1 | |
| | December | 1 | 1 | |
| 2018 | February | 3 | 3 | |
| | April | 4 | 1 | 3 |
| | May | 2 | 1 | 1 |
| | June | 1 | | 1 |
| | September | 5 | | 5 |
| | October | 3 | | 3 |
| | November | 1 | | 1 |
| 2019 | January | 2 | 1 | 1 |
| | February | 1 | | 1 |
| | March | 3 | | 3 |
| | April | 2 | | 2 |
| | May | 3 | | 3 |
| | June | 3 | | 2 |
| | July | 2 | | 2 |
| | August | 3 | | 3 |
| | September | 4 | | 4 |
| | October | 3 | | 3 |
| | November | 2 | | 2 |
| | December | 2 | | 2 |
| 2020 | January | 3 | | 3 |
| | February | 4 | | 4 |
| | March | 4 | | 4 |
| | September | 5 | 2 | 3 |
| | October | 38 | 32 | 6 |
| | November | 53 | 53 | |
| | December | 10 | 8 | 2 |
| 2021 | January | 9 | 9 | |
| | February | 11 | 11 | |
| | March | 21 | 20 | 1 |
| | May | 3 | 3 | |

| | | | | |
|---|---|---|---|---|
| | June | 11 | 11 | |
| | July | 5 | 5 | |
| | August | 6 | 5 | 1 |
| | September | 12 | 12 | |
| | October | 18 | 18 | |
| | November | 16 | 16 | |
| | December | 9 | 9 | |
| 2022 | January | 5 | 3 | 2 |
| | February | 2 | 1 | 1 |
| | March | 28 | 20 | 8 |

Note: serotypes of 354 strains isolated between April 2017 and March 2022 from Bangladesh that were tested. All tested strains isolated in 2017 were Inaba, as BD-2 strains. From April 2018, Ogawa serotype strains were identified as BD-1.2 strains. From September 2020, Inaba serotype strains of BD-1.2 were detected.

**Supplementary Table 6. Serotype and ctxB genotype profiles of randomly chosen strains isolated from clinical samples in Dhaka, Bangladesh, between March and September 2022**

| SL. | Strain ID | Date | Serotype | *ctx*B-type | Antibiotic Resistance Profile |
|---|---|---|---|---|---|
| 1 | DVC - 638 | 1/3/22 | Ogawa | B7 | IPM, SXT, AMP |
| 2 | DVC - 640 | 1/4/22 | Inaba | B7 | IPM, SXT, AMP |
| 3 | DVC - 642 | 1/5/22 | Inaba | B7 | SXT, AMP |
| 4 | DVC - 644 | 1/9/22 | Inaba | B7 | IPM, SXT, AMP |
| 5 | DVC - 646 | 1/10/22 | Ogawa | B7 | IPM, SXT, AMP |
| 6 | DVC - 650 | 2/6/22 | Inaba | B7 | SXT |
| 7 | DVC - 652 | 2/6/22 | Ogawa | B7 | SXT, AMP |
| 8 | DVC - 661 | 3/7/22 | Inaba | B7 | IPM, SXT, AMP |
| 9 | DVC - 663 | 3/7/22 | Ogawa | B7 | IPM, SXT, AMP |
| 10 | DVC - 664 | 13/3/2022 | Ogawa | B7 | IPM, SXT |
| 11 | DVC - 667 | 14/3/2022 | Inaba | B7 | AMP |
| 12 | DVC - 670 | 27/3/2022 | Inaba | B7 | IPM, SXT, AMP |
| 13 | DVC - 681 | 4/5/22 | Ogawa | B7 | IPM, SXT, AMP, AMC |
| 14 | DVC - 685 | 19/4/2022 | Inaba | B7 | IPM, SXT, AMP |
| 15 | DVC - 687 | 24/4/2022 | Inaba | B7 | IPM, SXT, AMP |
| 16 | DVC - 689 | 5/10/22 | Inaba | B7 | SXT, AMP, AMC |
| 17 | DVC - 695 | 16/5/2022 | Inaba | B7 | IPM, SXT, AMP |
| 18 | DVC - 696 | 17/5/2022 | Ogawa | B7 | IPM, SXT, AMP |
| 19 | DVC - 697 | 17/5/2022 | Inaba | B7 | SXT, AMP, AMC |
| 20 | DVC - 698 | 22/5/2022 | Ogawa | B7 | IPM, SXT, AMP |
| 21 | DVC - 700 | 29/5/2022 | Inaba | B7 | IPM, SXT, AMP |
| 22 | DVC - 702 | 6/8/22 | Ogawa | B7 | IPM, SXT, AMP |
| 23 | DVC - 705 | 20/6/2022 | Inaba | B7 | IPM, SXT, AMP, AMC |
| 24 | DVC - 706 | 20/6/2022 | Ogawa | B7 | IPM, SXT, AMP |
| 25 | DVC - 707 | 28/7/2022 | Ogawa | B7 | IPM, SXT, AMP |
| 26 | DVC - 709 | 8/8/22 | Ogawa | B7 | IPM, SXT, AMP, AMC |
| 27 | DVC - 712 | 21/8/2022 | Ogawa | B7 | IPM, SXT, AMP, AMC |
| 28 | DVC - 715 | 9/6/22 | Ogawa | B7 | IPM, SXT, AMP |
| 29 | DVC - 719 | 19/9/2022 | Ogawa | B7 | IPM, SXT, AMP |
| 30 | DVC - 723 | 27/9/2022 | Ogawa | B7 | IPM, SXT, AMP |

These 30 randomly chosen strains were isolated during and after 2022 massive cholera outbreak in Bangladesh, but they were left out of the sequencing and phylogenetic analysis. According to the ctxB genotype, the randomly chosen strains are likely to belong to the lineage of Asian clade, namely to BD-1.2. Drug Tested - ampicillin (AMP, 10 μg), cefuroxime (CXM, 30 μg), ciprofloxacin (CIP, 5 μg), mecillinam (MEL, 25μg), erythromycin (E, 15 μg), imipenem (IMP, 10 μg), sulfamethoxazole/trimethoprim (SXT, 25 μg), tetracycline (TE, 30 μg), aztreonam (ATM, 30 μg), azithromycin (AZM, 15 μg), chloramphenicol (C, 30 μg), and gentamicin (CN, 10 μg).

## Supplementary References


1. Li, H. & Durbin, R. Fast and accurate short read alignment with Burrows–Wheeler transform. *Bioinformatics* **25**, 1754–1760 (2009).
2. Li, H. *et al.* The Sequence Alignment/Map format and SAMtools. *Bioinformatics* **25**, 2078–2079 (2009).
3. Li, H. A statistical framework for SNP calling, mutation discovery, association mapping and population genetical parameter estimation from sequencing data. *Bioinformatics* **27**, 2987 (2011).
4. Quick start - BLAST® Command Line Applications User Manual - NCBI Bookshelf. https://www.ncbi.nlm.nih.gov/books/NBK569856/.
