# Supplementary material for: Genomic attributes of Vibrio cholerae O1 responsible for 2022 massive cholera outbreak in Bangladesh
Source: Nat Commun. 2023 Mar 1;14:1154. doi: 10.1038/s41467-023-36687-7 (PMC9977884; doi:10.1038/s41467-023-36687-7)
Supplement: Supplementary file 3 — Description to Additional Supplementary Information [file 41467_2023_36687_MOESM3_ESM.pdf]

Description of Additional Supplementary Files

Supplementary Data 1. List of V. cholerae strains used in this study

Supplementary Data 2. Lineageassociated SNPs clusters were identified using Hierarchical clustering. Here, A1-A3 are subclusters of a large cluster A, and B1- B5 are sub-clusters of a large cluster of B. SNPs of subcluster A1 were common for all lineages of the global clade, but not in the Asian clade. SNPs of A2 and A3 subclusters were acquired by different lineages of the global clade. SNPs of subcluster B1 were found in only the Asian clade. SNPs of subclusters B2-B5 were common between recent ancestral and descendent lineages global clade.

Supplementary Data 3. Genetic characteristics of the representative strains belonging to the Global and Asian clades.
