# Supplementary material for: Genomic attributes of Vibrio cholerae O1 responsible for 2022 massive cholera outbreak in Bangladesh
Source: Nat Commun. 2023 Mar 1;14:1154. doi: 10.1038/s41467-023-36687-7 (PMC9977884; doi:10.1038/s41467-023-36687-7)
Supplement: Supplementary file 7 — Reporting Summary [file 41467_2023_36687_MOESM7_ESM.pdf]

nature portfolio

Corresponding author(s): Dr. Munirul Alam

Last updated by author(s): 2023/02/08

Reporting Summary

Nature Portfolio wishes to improve the reproducibility of the work that we publish. This form provides structure for consistency and transparency in reporting. For further information on Nature Portfolio policies, see our Editorial Policies and the Editorial Policy Checklist.

Statistics

For all statistical analyses, confirm that the following items are present in the figure legend, table legend, main text, or Methods section.

n/a	Confirmed	
☐	☒	The exact sample size (*n*) for each experimental group/condition, given as a discrete number and unit of measurement
☒	☐	A statement on whether measurements were taken from distinct samples or whether the same sample was measured repeatedly
☐	☒	The statistical test(s) used AND whether they are one- or two-sided<br>*Only common tests should be described solely by name; describe more complex techniques in the Methods section.*
☒	☐	A description of all covariates tested
☒	☐	A description of any assumptions or corrections, such as tests of normality and adjustment for multiple comparisons
☐	☒	A full description of the statistical parameters including central tendency (e.g. means) or other basic estimates (e.g. regression coefficient) AND variation (e.g. standard deviation) or associated estimates of uncertainty (e.g. confidence intervals)
☐	☒	For null hypothesis testing, the test statistic (e.g. *F*, *t*, *r*) with confidence intervals, effect sizes, degrees of freedom and *P* value noted<br>*Give P values as exact values whenever suitable.*
☐	☒	For Bayesian analysis, information on the choice of priors and Markov chain Monte Carlo settings
☒	☐	For hierarchical and complex designs, identification of the appropriate level for tests and full reporting of outcomes
☐	☒	Estimates of effect sizes (e.g. Cohen's *d*, Pearson's *r*), indicating how they were calculated

Our web collection on statistics for biologists contains articles on many of the points above.

Software and code

Policy information about availability of computer code

Data collection	Publicly available sequence data were collected from ENA database using bash command.
Data analysis	The data was analyzed using bash scripts, python-3.9.13, and R scripts, and with the following softwares: fastp v0.23.2, Spades v3.15.4, ragout v2.3, Prokka v1.14.5, ResFinder, ABRicate v1.0.1, IQ-TREE v2.2.0, ModelFinder, BEAST v.2.6.7, PANNZER, GSEA-Pro.v3, REVIGO, Samtools v1.9, Bcftools v1.9, and blastn v2.13.0. In addition, in house scripts used for analysis is available in GitHub (https://github.com/mamunmonir/Vibrio_genomics).

For manuscripts utilizing custom algorithms or software that are central to the research but not yet described in published literature, software must be made available to editors and reviewers. We strongly encourage code deposition in a community repository (e.g. GitHub). See the Nature Portfolio guidelines for submitting code & software for further information.

Data

Policy information about availability of data

All manuscripts must include a data availability statement. This statement should provide the following information, where applicable:

- Accession codes, unique identifiers, or web links for publicly available datasets
- A description of any restrictions on data availability
- For clinical datasets or third party data, please ensure that the statement adheres to our policy

Newly sequenced data used in this study were submitted under Bio-project accessions IDs PRJDB13928 and PRJDB13857. Publicly available sequence data used in

this study was downloaded from the European Nucleotide Archive (ENA), and metadata along with accession numbers were given in Supplementary Data S1. Reference sequences for N16961 strain were downloaded from NCBI database (accession ID: NC_002505.1 and NC_002506.1). In addition, other relevant data were given in supplementary data, supplementary tables, and source data files.

Human research participants

Policy information about studies involving human research participants and Sex and Gender in Research.

Reporting on sex and gender	n/a
Population characteristics	n/a
Recruitment	n/a
Ethics oversight	n/a

Note that full information on the approval of the study protocol must also be provided in the manuscript.

Field-specific reporting

Please select the one below that is the best fit for your research. If you are not sure, read the appropriate sections before making your selection.

☒ Life sciences ☐ Behavioural & social sciences ☐ Ecological, evolutionary & environmental sciences

For a reference copy of the document with all sections, see nature.com/documents/nr-reporting-summary-flat.pdf

Life sciences study design

All studies must disclose on these points even when the disclosure is negative.

Sample size	In this study, we focused on the source attributes of the cholera bacterium associated with the massive outbreak which broke out surpassing all past records of the daily patients. To our knowledge, there has been no statistical approach for choosing appropriate number of strains for comparative genomic studies to understand the causal agent and the source attributes. Previous studies suggest, a small number of strains could be instructive for identifying source attribute. For example, to identify source of the Haitian cholera outbreak only 23 V. cholerae strains were used that suggested the Asian origin for the bacterium (Chin et al. 2011, N Engl J Med, DOI: 10.1056/NEJMoa1012928). Follow-up molecular typing and genomic studies on more V. cholerae strains isolated in Haiti upheld the conclusions made earlier by Chin et al. (2011) (Hendriksen et al., 2011, mBio, DOI: 10.1128/mBio.00157-11; Reimer et al., 2011, DOI:10.3201/eid1711.110794). Likewise, a recent study sequenced 42 strains to reveal the source attributes of the outbreak strains in Yemen where over 1.1 million cases and 2,300 deaths were reported during 2016-2017 (Weill et al., Nature, 2019; DOI: 10.1038/s41586-018-0818-3). We have sequenced 32 V. cholerae O1 El Tor strains that included 21 strains isolated from patients admitted at icddr,b hospital during the ongoing 2022 massive cholera outbreak, and compared with 949 El Tor genome sequences from 88 countries covering the period 1957 - 2021 (Total n=981). In addition, another 30 strains isolated between March and September 2022 from the clinical sample of the patients admitted at icddr,b hospital, Bangladesh, were tested for serotype, ctxB genotype, and drug resistance to increase the power of the study concluded. This study did not need participant consent since there was no risk involved, no personally identifiable information or identifiable biospecimens were collected, and no follow-up was made after collecting the stool samples.
Data exclusions	No data were excluded from the analyses
Replication	In this, there were no experimental discoveries. Thus replication is not required.
Randomization	Dipstick positive stool samples were collected randomly from the patients admitted at icddr,b hospital. Then a subset of samples were randomly selected for sequencing and typing for ctxB, and drug sensitivity at our laboratory.
Blinding	Individual level information of the patients or any other characteristics were not considered for selecting samples for sequencing and typing.

Reporting for specific materials, systems and methods

We require information from authors about some types of materials, experimental systems and methods used in many studies. Here, indicate whether each material, system or method listed is relevant to your study. If you are not sure if a list item applies to your research, read the appropriate section before selecting a response.

Materials & experimental systems

n/a	Involved in the study
☒	☐ Antibodies
☒	☐ Eukaryotic cell lines
☒	☐ Palaeontology and archaeology
☒	☐ Animals and other organisms
☒	☐ Clinical data
☒	☐ Dual use research of concern

Methods

n/a	Involved in the study
☒	☐ ChIP-seq
☒	☐ Flow cytometry
☒	☐ MRI-based neuroimaging
